# Advances in clinical trial design for development of new TB treatments—Translating international tuberculosis treatment guidelines into national strategic plans: Experiences from Belarus, South Africa, and Vietnam

**DOI:** 10.1371/journal.pmed.1002896

**Published:** 2019-10-18

**Authors:** Grania Brigden, Nguyen Viet Nhung, Alena Skrahina, Norbert Ndjeka, Dennis Falzon, Matteo Zignol

**Affiliations:** 1 Department of Tuberculosis, The International Union Against TB and Lung Disease, Geneva, Switzerland; 2 National Lung Hospital, Vietnam NTP, Vietnam; 3 Republican Scientific and Practical Centre for Pulmonology and TB, Minsk, Belarus; 4 Drug-Resistant TB, TB and HIV directorate, National Department of Health, Pretoria, South Africa; 5 Global Tuberculosis Programme, World Health Organization, Geneva, Switzerland

**Summary points**

- The World Health Organization (WHO) plays an important role in setting global norms and standards with a focus on public health and publishes international guidelines regularly to support Member States, particularly ministries of health, in the provision of the highest standard of healthcare.
- Over the last 5 years, multiple advances in diagnosis and treatment of tuberculosis (TB) have resulted in a number of new WHO guidelines for TB care, but these recent guidelines have not always been implemented in a timely fashion, raising issues in their adoption and scale-up at country level.
- We discuss the experiences of three countries with a high burden of multidrug-resistant TB (MDR-TB)—Belarus, South Africa, and Vietnam—in implementing recent WHO guidelines on bedaquiline, a drug recently registered and recommended for the treatment of MDR-TB and the standardised shorter treatment regimen (STR) for MDR-TB.
- The process of adopting and implementing new guidelines requires national TB programmes (NTPs) to interact with multiple agencies: both intergovernmental departments and external agencies such as regulators and donors. These processes are country specific, but there are some generalised challenges that NTPs in high-burden countries experienced when implementing recent WHO MDR-TB guidance.
- With multiple trials of new regimens for MDR-TB and new classes of drugs in the clinical treatment pipeline, the frequency of new guidelines for TB is expected to increase, and it is important to support NTPs to implement and scale-up these new developments in treatment.

## Introduction

One of the key missions of national tuberculosis (TB) programmes (NTPs) is to issue policy and technical guidance for clinicians and healthcare workers involved in TB care at the country level. These national policies are generally developed based on international public health guidelines, such as those issued by the World Health Organization (WHO) [1, 2].

Updating national policies or technical guidelines in view of recent advances in TB diagnosis, care, and prevention has an important impact on TB patients, the health system, the community and is key to ensuring the best quality of care for people with TB.

WHO has a mandate to provide technical assistance to its Member States on different aspects of public health. The 13th General Programme of Work of WHO [3] outlines the organisation’s status as a science- and evidence-based agency setting global norms and standards, with a focus on public health. Translating research findings into policies may be a challenging task, given that the design of clinical studies may not always address the main public health priority directly, and recommended interventions require substantial adaptation to the particular programme conditions and settings [4].

In 2007, WHO established the Guideline Review Committee (GRC) to provide oversight to organisational efforts to ensure that policy guidance is up-to-date, trustworthy, feasible, and developed in a transparent way, in line with the highest international standards of care [5], and adheres to WHO principles for policy development [6]. The WHO-convened Guideline Development Group advises on the scope of the guidelines, assesses the quality of available evidence, and formulates recommendations using a systematic method termed Grading of Recommendations Assessment, Development, and Evaluation (GRADE) [7]. This approach requires experts who are formulating recommendations to base their judgements not only on trial evidence but also on other considerations, such as the balance of expected desirable and undesirable effects, equity, resource use, feasibility, and acceptability to the populations targeted by the guidance. These changes have contributed to an improvement in purpose, clarity, and the methodological quality of WHO guidelines in the last decade [7].

The pace of developments in new TB diagnostics, treatment, and patient support has increased substantially over the last decade, leading to the release of over 20 new or updated WHO guidelines on different aspects of TB care since 2010 [8]. This pace is expected to continue, and the *PLOS Medicine* Collection of which this paper is part [9] discusses the optimal characteristics of clinical trial designs to inform future policy guidance for new TB regimens.

Already in the last 5 years, NTPs have had to respond to a number of WHO policy updates on multidrug-resistant TB (MDR-TB) treatment as new medicines became available and results from studies on the use of novel drugs and the standardised shorter treatment regimen (STR) were communicated (e.g., bedaquiline, delamanid, and the 9–12-month-shorter MDR-TB regimen) [10–16]. Partly as a result of these rapid changes, a number of these new treatment policies have not been adopted or fully implemented by national programmes. A recent review [17] of national policies in 29 countries highlighted national policy gaps when compared to WHO policies. Thus, in the case of WHO’s recommended 9–12-month-shorter MDR-TB regimen, 45% of the countries had developed policies, but only 69% of those countries had implemented them. By the end of 2017, 62 countries, mostly in Africa and Asia, reported having used shorter MDR-TB regimens; between 2016 and 2017, the number of patients reported to have been started on the 9–12-month-shorter regimen globally increased from 2,400 to 10,000 [18]. With regard to the new drugs, bedaquiline and delamanid, 86% of countries had a policy on bedaquiline and 67% on delamanid, but the actual use of the new drugs reflected the implementation gap, with only 12,194 and 976 treatment courses procured globally for bedaquiline and delamanid, respectively, in 2017 [19].

There are multiple barriers to the adoption of international treatment guidelines, including factors relating to the acceptability and perceived feasibility of the recommendation, the individual opinion of clinicians, patient preferences, regulatory processes for new drugs, requirement for new resources, and the financial and political commitment from the Ministry of Health (MOH) [20].

The following case studies from the NTPs of three high-burden countries refer to national experiences in the introduction of new drugs and regimens for MDR-TB to illustrate how countries approached implementation of new policies for TB treatment. Belarus, South Africa, and Vietnam are all on WHO’s high-burden MDR-TB list but with different epidemic patterns (see Table 1). The case studies review the experiences of the countries in implementing the interim guidance for the use of bedaquiline in the treatment of MDR-TB, issued by WHO in 2013 [21], and the revised guidelines on treatment of MDR-TB issued in 2016 that recommend the use of the 9–12-month-shorter MDR-TB regimen under certain conditions [13].

**Table 1:** Overview of the TB epidemic in Belarus, South Africa, and Vietnam [18].

Indicator	Belarus	South Africa	Vietnam
Total population (2017)	9.5 million	57 million	96 million
TB prevalence (all forms)	32.1 per 100,000	398.6 per 100,000	110.1 per 100,000
TB incidence (new and relapse cases)	29.3 per 100,000	386.3 per 100,000	107.0 per 100,000
HIV prevalence among TB	2.9 per 100,000	340 per 100,000	4.7 per 100,000
Incidence of MDR/RR-TB	26 per 100,000	25 per 100,000	7.4 per 100,000
Percent of new cases with MDR/RR-TB	38% (36–41)	3.4% (2.5–4.3)	4.1% (2.8–5.7)
Percent of retreated cases with MDR/RR-TB	67% (63–70)	7.1% (4.8–9.5)	17% (17–18)
TB treatment coverage	80%	68%	83%
MDR/RR-TB treatment success rate (2015)	64% (cohort size: 1,400)	55% (cohort size: 9,750)	74% (cohort size: 2,045)
XDR-TB treatment success rate (2015)	53% (cohort size: 508)	48% (cohort size: 427)	Not reported

Abbreviations: MDR/RR-TB, multidrug-resistant/rifampicin-resistant TB; TB, tuberculosis; XDR-TB, extensively drug-resistant TB

### Implementation of bedaquiline in Belarus

In 2017, there were an estimated 3,500 new TB cases in Belarus of which 2,500 had rifampicin resistance or MDR-TB [18]. In 2012, in anticipation of the approval of a new drug for TB, WHO released a handbook to advise countries on how to organise both spontaneous and active pharmacovigilance [22]. The national pharmacovigilance centre of the Belarus MOH, with its prior experience in active pharmacovigilance in the country for antiretrovirals [23], established strong links with the NTP to enhance pharmacovigilance among MDR-TB patients. The implementation of cohort event monitoring for MDR-TB treatment on regimens containing linezolid, and later bedaquiline, were labour-intensive activities for MOH staff, undertaken without additional resources [24] (Table 2).

**Table 2:** Key milestones in the successful introduction of new medicines for MDR-TB patients, Belarus.

Actions to strengthen MDR-TB treatment	Actions to strengthen patient safety
• Aligning national TB guidelines to WHO recommendations • Training of clinical staff in the new policies and in case management • Piloting and subsequent scale-up of video-supported therapy as an adjunct to patient-centred care • Strengthening of laboratory capacity to detect drug resistance using newer techniques and to perform increasing volumes of culture • Changes in the drug procurement system, including ministerial waiver for the importation of new medicines • Updated national electronic TB register to include information on adverse events and details on regimen • Funding proposal to the Global Fund to provide financial resources • Technical support provided by WHO and by Médecins sans Frontières	• 2012: Links cultivated between national TB programme and the NPV • 2012: NPV strengthens its methods for both spontaneous reporting and for active surveillance (using CEM) • 2012: CEM for antiretroviral treatment starts • 2013: CEM for antiretroviral treatment extended to patients with HIV who had MDR-TB • 2014: CEM for linezolid-containing regimens started in MDR-TB • 2016: aDSM introduced for all MDR-TB patients on treatment • 2017: aDSM data reported to global database

Abbreviations: aDSM, active TB drug safety monitoring and management; CEM, cohort event monitoring; MDR-TB, multidrug-resistant TB; NPV, national pharmacovigilance centre; TB, tuberculosis; WHO, World Health Organization

In mid-2013, the national TB guidelines were updated in alignment with the new WHO policy on bedaquiline use (including translation into the Russian language) and staff training organised by the MOH under the guidance of the MDR-TB expert group (consilium). The MDR-TB consilium is a platform of multidisciplinary experts from Belarus with the aim to improve the quality of diagnosis and care and to reduce the time to initiation of effective MDR-TB treatment throughout the country. The NTP also benefited from reviews of its work by WHO, Médecins sans Frontières, the Supranational Reference Laboratory, and other external experts. Measures were taken by the Council of Ministers to waive the requirements for drug registration for bedaquiline. The same mechanism was used subsequently to permit the use of other medicines, including clofazimine and delamanid. The support of the Global Fund was critical in securing resources to purchase bedaquiline and the companion medicines. By October 2018, 543 patients had started treatment with bedaquiline. In 2018, Belarus reported individual case-based data from programmatic cohorts of patients treated with bedaquiline-containing regimens to the pooled analysis for the latest update of WHO’s MDR-TB treatment guidelines [25, 26]. An important challenge faced by the MOH when implementing bedaquiline was for healthcare staff to adhere to proper criteria when selecting patients to be placed on regimens including this new agent. The MDR-TB expert consilium played an important role to ensure compliance. Another limitation was to have all the medicines needed for the regimen available at the time of start of treatment: this required coordination with all stakeholders (i.e., funders, logistics, facilities) to limit delays. The WHO-recommended 9–12 month STR MDR-TB regimen in Belarus is contraindicated in many because MDR-TB patients commonly have strains harbouring additional resistance to pyrazinamide and to key second-line drugs such as fluoroquinolones and injectable agents. This is why the focus has been on scaling up the use of bedaquiline, with other second-line drugs that have not been previously used in Belarus. Since late 2018, the NTP introduced under operational research conditions a shorter regimen of 9 months consisting of all group A and B medicines recommended in MDR-TB regimens.

In 2015, following WHO advice on active TB drug safety monitoring and management (aDSM) in patients treated with novel regimens and repurposed medicines [27], Belarus became an early adopter of aDSM as a standard of care and among the first countries to contribute records to WHO’s global aDSM database [28]. Using domestic and external funding, the Belarus MOH is updating the national electronic TB patient register to enhance future data management.

The articulated response from the MOH, including strengthening the surveillance and preventive and curative components of the NTP [29], has resulted in high case detection of TB, TB/HIV, and drug-resistant TB and treatment success in new and relapsed TB patients approaching 90% [30].

### Introducing bedaquiline in South Africa

South Africa is a country with high TB, MDR-TB, and HIV burden. The country contributes approximately 10% of global MDR-TB cases diagnosed and reported, with treatment success similar to the global rate at 54% and mortality at just above 20% [18].

**Fig 1:**
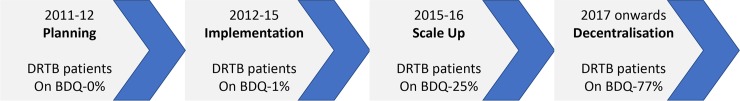
South African implementation pathway for BDQ. BDQ, bedaquiline; DRTB, drug-resistant tuberculosis.

The use of bedaquiline in the country started in December 2012, when the South Africa Medicines Control Council (MCC) approved the drug as part of a clinical access programme [31]. The programme was implemented at five sites and was later scaled up to 12 sites in 2014 after early successful results were obtained [32]. Once bedaquiline received full registration with the MCC, the inclusion criteria were broadened, and from 2017, bedaquiline use was decentralised to the district level to facilitate scale-up (Fig 1). In June 2018, South Africa announced that bedaquiline would be available to all eligible patients with rifampicin resistance, replacing the injectable agents in both the recent WHO-recommended longer treatment regimens as well as variants of the STR [26]. The STR has been included in national policies since 2015 [33], but similar to Belarus, the eligibility criteria for the STR have meant that its use has been limited in a population with increasingly complex resistance patterns. However, since September 2018, the South African NTP recommended a modified injectable-free STR nationwide. This regimen has the addition of linezolid for 2 months, with bedaquiline replacing the injectable agent and given for 6 months and levofloxacin replacing moxifloxacin [34].

The primary challenge to adoption and implementation of bedaquiline use has been the full regulatory approval required from the MCC, as the initial approval was only for a compassionate-use programme. The process to reach full regulatory approval took 18 months. Once registered, there was hesitancy of clinicians on the use of a new drug for which programmatic data were initially extremely limited. Subsequently, data were collected from pilot sites and published. A National Clinical Advisory Committee was formed to support implementation of WHO guidance by helping physicians design effective treatment regimens and establishing provincial committees to discuss difficult clinical cases. The NTP discussed WHO guidelines with local researchers and academia to ensure the guidance was customised to the national context and translated into practice. An additional challenge to the scale-up was maintaining a secure supply of stocks, particularly as bedaquiline was not on national tender.

### Improving diagnosis and treatment of MDR-TB in Vietnam

Vietnam is one of the 20 countries considered to have both a high TB and MDR-TB burden [18]. In 2016, Vietnam had 106,527 registered cases of TB, and it is estimated that 20% of cases are not detected [18]. To address this problem, the NTP developed the 2X strategy (Xray-Xpert MTB/RIF) to enhance early TB and MDR-TB detection. This strategy, in line with WHO guidance on the use of Xpert MTB/RIF [35–36] and chest radiography [37], aims to screen for and confirm TB infection and disease, including rifampicin resistance status, at the start of treatment.

**Fig 2:**
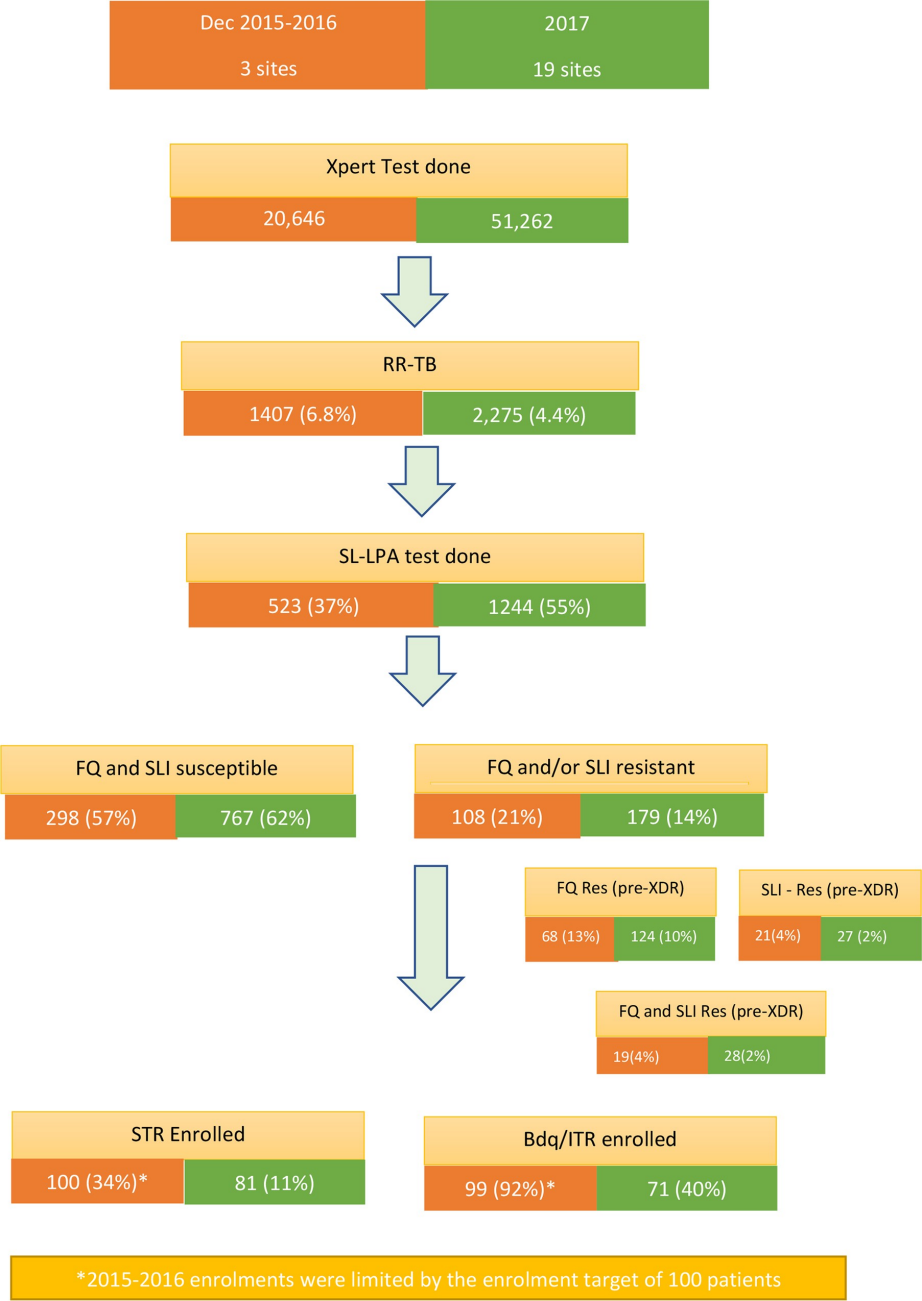
Patient triage approach in Vietnam. Bdq, bedaquiline; FQ, fluoroquinolone; ITR, individualised treatment regimen; Res, resistance; RR-TB, rifampicin-resistant tuberculosis; SLI, second-line injectable; SL-LPA, second-line–line probe assay; STR, standardised shorter treatment regimen; XDR, extensively drug resistant.

The scale-up of newer diagnostics was coupled with a patient triage strategy with bedaquiline and the STR part of the strategy. As clofazimine, a key drug in the shorter regimen and a companion drug to bedaquiline, was not registered in the country, the NTP had to apply for an investigation study to be approved by the institutional review board of the MOH so as to allow importation of the drugs needed. Bedaquiline was introduced under import waiver in December 2015 with the shorter treatment regimen introduced in April 2016, in three pilot provinces, and with the implementation for the STR expanded to an additional eight provinces after 18 months [38]. The expansion occurred after WHO’s recommendations on the short-course regimen in 2017 [14]. During this stepwise scale-up of the use of bedaquiline and the STR, the scale-up was interrupted because of a 7-month interruption pending MOH approval of the expansion. During this time, the STR enrolment declined from 32% to 11%; and bedaquiline use in those eligible declined from 92% to 40% (Fig 2). Following these pilots, the STR was included in the national guidance in 2018 and is now a major treatment option for MDR-TB countrywide.

**Fig 3:**
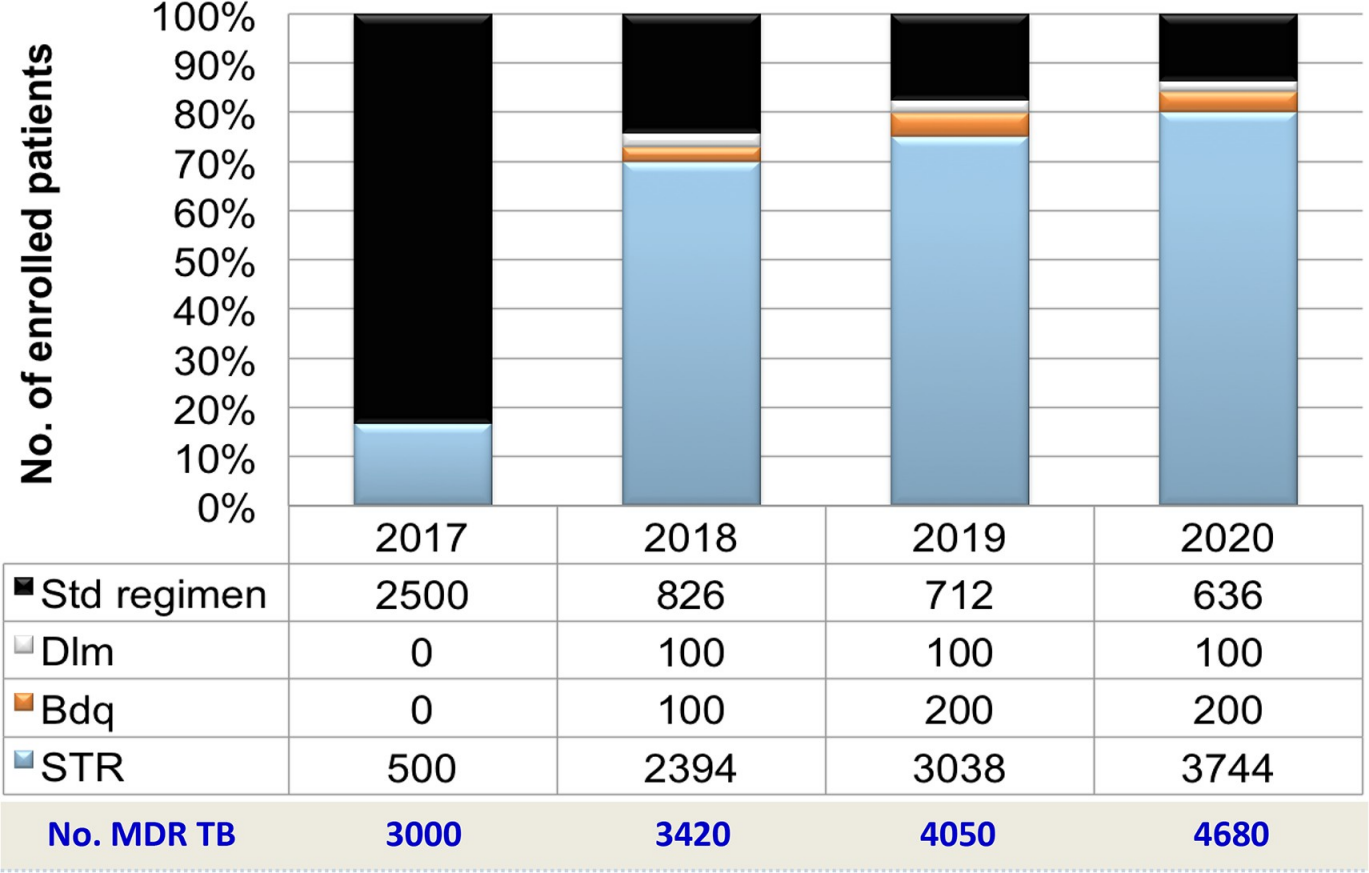
Scale-up plans for STR and Bdq in Vietnam. Bdq, bedaquiline; Dlm, delamanid; MDR TB, multidrug-resistant tuberculosis; Std, standard; STR, standardised shorter treatment regimen.

The long-term plan in Vietnam is to continue to scale up the use of bedaquiline. Based on local cohort studies, laboratory capacity was available to identify susceptibility of almost all drugs before indication of the regimen for individual patients, and the Vietnam NTP decided to apply modified STR as the primary regimen to treat drug-resistant TB. The planned stepwise scale-up of the modified shorter treatment regimen for drug-resistant TB treatment is shown in Fig 3. In order to overcome challenges regarding drug importation for bedaquiline, the drug has now been registered in 2019 for compassionate use while the main regulatory process is underway.

Policy change in Vietnam requires a stepwise approach, utilising pilot projects with scale-up happening over a 3–4-year timeline. At the same time of implementing pilot projects, the NTP negotiates in-country drug registration processes. The involvement of the WHO country office with technical assistance and support for policy change has helped to minimise delays in these processes.

## Discussion

WHO guidance strives to make recommendations that are based on the best and latest available evidence and that have applicability to diverse settings worldwide. The use of standardised evaluation methods like GRADE aims to assess study findings in a rigorous way but also ensure that due considerations for implementation are being addressed. However, WHO’s guideline processes cannot consider the nuances and sensitivities of the local socioeconomic, regulatory, and cultural conditions—this is left to the NTP when reviewing the guidance. As shown in the case studies described here, translating the research findings underlying new WHO guidance into programmatic guidance incurs substantial logistical challenges and delays for NTPs to mobilise the necessary resources and negotiate the regulatory framework. As in the three country examples, the process of adapting the recent WHO guidance on bedaquiline to the national situation is a multistage process, involving actors outside the NTP, such as donors and regulatory authorities, and is prone to delays.

**Fig 4:**
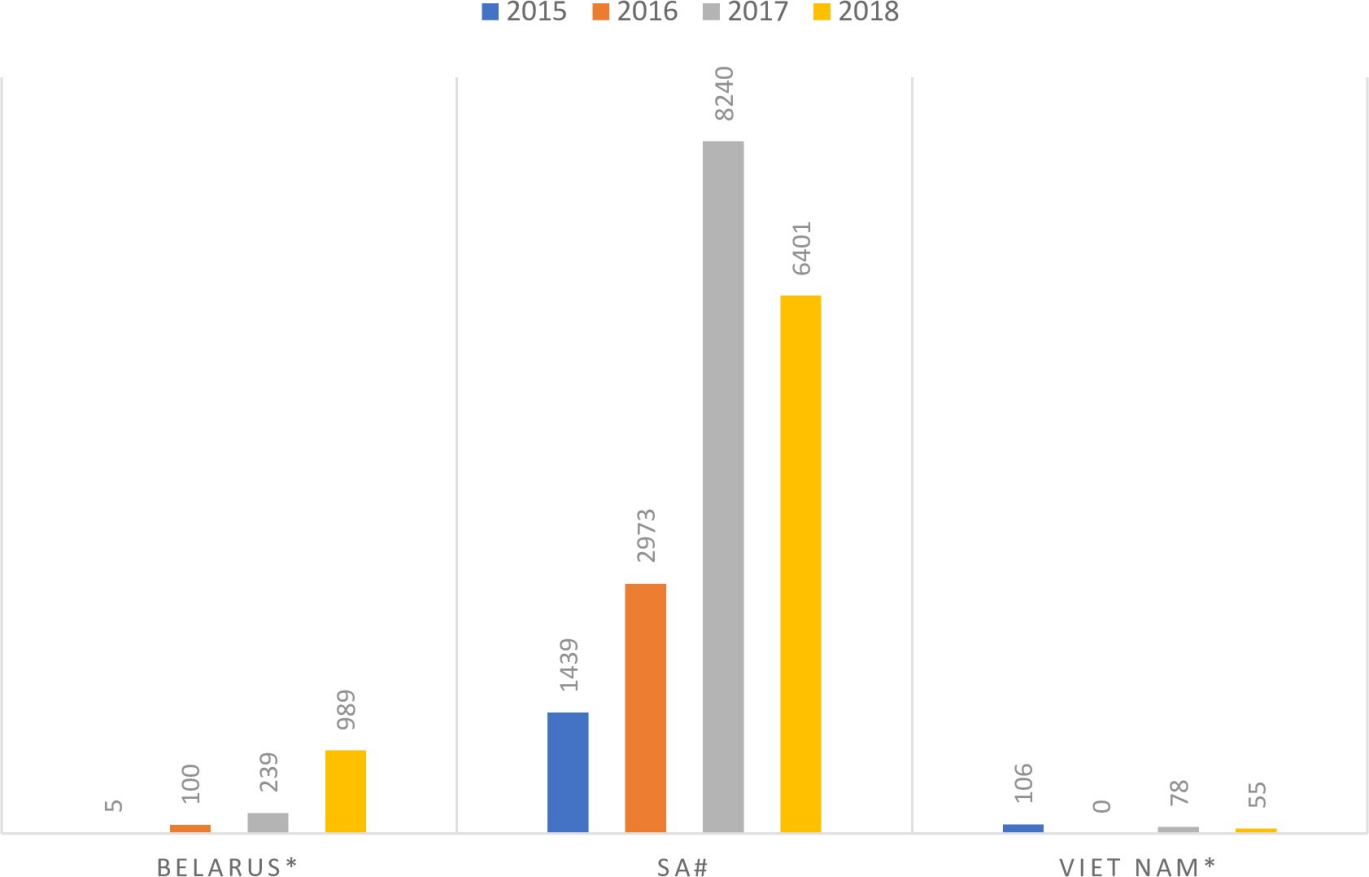
Scale-up of bedaquiline (WHO guidance issued in June 2013). SA, South Africa; WHO, World Health Organization.

The case studies highlight the challenges of introducing a new drug, particularly one with limited data on effectiveness and no long-term outcome data. The NTPs had to complete the necessary ethical, surveillance, and regulatory processes, and often pilot projects had to be undertaken to obtain real-life experience in the country, delaying the scale-up of the new drug (see Fig 4).

At the same time as new drugs were recommended to be added to the longer individualised regimen, WHO recommended a shorter standardised regimen for certain types of MDR-TB. NTP managers and staff had to work out how to implement the new drugs into their programmes as well as into a new treatment regimen, and this often required collecting data on efficacy and safety of both a new drug and a new regimen. Similarly, they had to ensure necessary funding not only to support the policy change process but also to procure the new drugs and the components of the standardised regimen, implement robust aDSM, and organise technical assistance or training for implementing the new policies. This required consideration of either national or donor resources, further adding to the implementation timeline, particularly for low- and middle-income countries that rely on the Global Fund and other donors to support their MDR-TB programmes. The recent update to the MDR-TB guidelines continues to recommend this dual approach of longer individualised regimens and more standardised shorter regimens [39].

To ensure that these new developments reach all relevant at-risk groups, the NTP needs to further engage with the national Ministry of Justice, Ministry of Migration, or other specific ministries. In countries that have placed TB high on the political agenda—such as Belarus, South Africa, and Vietnam—support for this engagement with other ministries may be easier than for other countries whose NTP may not have the support to engage with other ministries and national processes.

This policy update process needs to be repeated with the latest WHO guidance on MDR-TB [40], which has a number of significant changes for the NTP to consider. Bedaquiline scale-up and use will continue, as now bedaquiline is a group A drug (group A drugs are drugs that are strongly recommended for inclusion in a longer MDR-TB regimen) and as such is a key component of the new all-oral individualised long regimen [26]. The STR remains in the recommendations with a change in the injectable agent being used. With the welcome push for an all-oral regimen for MDR-TB, NTPs may want to consider operational research into the role of oral alternatives to the injectable agent in the STR, as has been done in South Africa, Belarus, and Vietnam. With another new drug, pretomanid [18], submitted for registration, and new regimens being recommended for latent TB infection (LTBI), the lessons learned implementing new or unregistered drugs and new regimens for MDR-TB will aid NTPs to ensure these new developments are adopted and scaled up, potentially using the pathways used for bedaquiline and the STR uptake.

## Conclusion

The experience of Belarus, South Africa, and Vietnam suggests that intergovernmental collaboration and new guideline adoption and implementation are facilitated when TB has been placed high on the political agenda, in contrast to other countries where TB maintains a much lower profile. The pathways and tools developed by NTPs to implement the new TB drugs and regimens for MDR-TB can help ensure that the latest WHO guidance on MDR-TB and LTBI can be implemented and scaled up quickly. With strengthened programmes (including implementation of aDSM), NTPs can generate the evidence to show whether new drugs and regimens found to be effective in clinical trials will work in populations that need them most [40].

With the TB drug and regimen pipeline at its healthiest in over a decade, advances in all areas of TB care are expected in the next decade requiring national guidelines to adapt as a priority. More updates to new guidance issued recently by WHO for the treatment of MDR-TB and LTBI are expected imminently as new drugs are submitted for registration, as well as results from new regimen studies being published in the coming years. A culture of change needs to be fostered and budgeted for and recognition needs to be given to countries that have supported their NTPs in this process. All actors in TB care, from international donors to national funding and regulatory agencies, need to support this approach to change, reacting promptly to and supporting new developments in TB therapeutics. The political attention to TB at the recent UN high-level meeting on TB [41] must be followed up with the appropriate funding and policy support so that NTPs are supported to rapidly review and adopt the best standard of care for people with TB. A systematic approach to evaluate how policies are used and adapted by countries and their impact—both as intended and inadvertent—would be a fruitful step in the feedback cycle that WHO and other professional bodies use when planning updates of new policy guidelines.

## Abbreviations

aDSM: active TB drug safety monitoring and management
FQ: fluroquinolone
GRADE: Grading of Recommendations Assessment, Development, and Evaluation
GRC: Guideline Review Committee
LTBI: latent TB infection
MCC: Medicines Control Council
MDR-TB: multidrug-resistant TB
MOH: Ministry of Health
NTP: national TB programme
RR-TB: rifampicin-resistant TB
SLI: second-line injectable
SL-LPA: second line–line probe assay
STR: standardised shorter treatment regimen
TB: tuberculosis
WHO: World Health Organization
XDR-TB: extensively drug-resistant TB
